# Medicine and Birds

**Published:** 1983-07

**Authors:** Philip Radford

**Affiliations:** General Practitioner (Rtd), Westbury-on-Trym


### Medicine and Birds

**Philip Radford**
General Practitioner (Rtd), Westbury-on-Trym

No doctor can escape some degree of association with birds in the course of his professional life. The general practitioner, as he examines yet another case of Salmonella food poisoning, might well ponder about this, particularly as he tells his patients that they have allowed insufficient time for the thawing out of a frozen chicken or turkey before its cooking. Considering the prevalence of Salmonella infection in poultry, perhaps it is surprising that food poisoning by this group of organisms is not more widespread than at present. Probably most family doctors would agree that the current situation represents a significant public health problem, even if the illness is usually a mild one; after all, it is only rarely that one encounters a really serious gastro-enteritis such as botulism and that, incidentally, has been caused by eating infected duck paste.

Advice and general hygiene are the important factors in preventing Salmonella food poisoning; contrary to what most patients seem to expect, antibiotics have no value in influencing the clinical course and do not help the carrier state. Turning now from poultry to wild birds, another potential source of Salmonella infection is from gulls, where droppings from affected birds may contaminate open reservoirs or rivers. Nowadays large numbers of gulls, mostly black-headed gulls *Larus ridibundus*, roost at inland reservoirs.

Then, while unconnected with gastro-enteritis, another hazard of a poultry meal is that splinters of bone may become lodged in the pharynx or oesophagus and, in the extreme case, perforation of the oesophagus may occur with resulting mediastinitis. I think that a patient with such a life-threatening disorder as this would readily admit that a bird had importance in the aetiology of his illness. These days a bone originating from a commercially reared chicken or turkey is the most likely offender but a trapped or shot wild goose or duck could well be implicated.

Rather than eating birds, those who keep them as pets can develop medical problems too. Just as some people become proud and possessive of their dogs or cats, so do others for their caged budgerigars or canaries. Many people derive psychological satisfaction from grooming their pets and providing food for them; some, especially the elderly or lonely, like to think that their pet is dependent on them and hope that, in turn, the pet is 'grateful' for the care it receives. The purring of a stroked cat is a welcome reward. Similarly, other people are rewarded by the songs or bright plumage of their birds although the outcome for a minority of these individuals is, rather cruelly, respiratory disease through bird-fancier's lung.

With this disorder the patient reacts to the organic dust which is produced on cleaning out the bird-cage; essentially, the lesion is an allergic alveolitis. To most of us, the base of a bird-cage is hardly an attractive place and it is not difficult to appreciate the feelings of a patient who opens a consultation with 'My mouth feels like the bottom of a bird-cage, doctor.' Such an expressive complaint, however, scarcely suggests the onset of respiratory disease caused by the onset of sensitivity to caged birds or their products.

The commonest bird species to give rise to bird-fancier's lung is the budgerigar, which is a member of the parrot family. Serum proteins become mixed with the birds' droppings, and it is these proteins to which the victim becomes sensitised. So if a patient develops attacks of breathlessness, especially with cyanosis and finger clubbing, it is as well to consider the possibility of a bird pet cause. Serum precipitin determination should help in the diagnosis; a specific antigen may be identified but, admittedly, this gives no more than proof of exposure.

Hopefully, in the early stages, if the budgerigar or parrot is removed, then the chest symptoms resolve. Sometimes it is worthwhile for the patient to attempt, experimentally, to keep another bird species such as a canary, which is a finch; this course may be successful as different proteins are involved. Understandably, should an affected patient happen to manage a pet-shop, there is nothing else he can do but change his occupation or deal in only a limited number of bird species which he knows, by trial and error, he is able to tolerate.

A possible danger from contact with pigeons, budgerigars or parrots is the infective disease of psittacosis. With this disorder, birds may be carriers of the psittacosis organism for a considerable length of time. The bird's original illness may not necessarily have been at all severe; infection is transferred by dust from the feathers or from the droppings. The human patient may have only a mild illness or, indeed, even a subclinical one, but fatal cases do occasionally occur. Fever, aching limbs and a pneumonic condition should alert the doctor to the possibility of the diagnosis in a bird keeper. Serum agglutinins for psittacosis will show a rising titre; happily, tetracycline is usually an effective treatment. Yes, birds both alive and dead do have a possible risk of infection for people but, apart from an infective process, some individuals are upset if they even come near a few feathers. A pillow stuffed with feathers, for example, is enough to precipitate bronchial asthma in a patient with such a hypersensitivity.

Probably most people in Britain, and certainly the majority of those with any interest in natural history, prefer to watch birds in the wild state rather than those in cages. One of the most familiar of woodland birds is the robin *Erithacus rubecula*; it has adapted, in Britain at least, to living near habitation and follows the gardener as the soil is turned. Some garden robins become very tame, especially in hard weather conditions, where householders regularly supply food scraps; occasionally, enthusiasts will provide irresistible items such as mealworms which may scare unprepared members of the family if the hoard is found unexpectedly. Such is the concern of some people for their resident robins, that I have known elderly and quite infirm patients who have insisted on digging the ground in frosty weather so that their birds might have access to earthworms.

Robins are inquisitive and often aggressive birds and, moreover, both sexes maintain territory and sing in autumn and winter. Not uncommonly, I have heard a patient describe himself as being 'as weak as a robin' and I have noticed that this description is used more by males than females. But, other than an injured bird, I have never watched a robin which was torpid or failed to be vigilant. I find it difficult to know how the phrase arose; it cannot be due entirely to the bird's size for patients do not suggest that they are weak like a wren *Troglodytes troglodytes* or a blue tit *Parus caeruleus*, both of which are common birds and smaller than the robin. It seems strange that post-influenzal debility should be referred to as a robin-like weakness.

Certainly there is no suspicion of weakness as a robin lunges at a partly-hidden wireworm or as it suddenly seizes a spider from a bark crevice. True, the song may be languid in its mode of delivery but there is great variation in the phrases; it is a pleasing, melodious warble which has a different character in autumn from that of the spring. A robin is always watchful in its territory; the resident bird puffs its red breast towards any intruder of the same species, while the body is swayed from side to side. As a result of this intimidation, the would-be aggressor usually retreats and flies off but, in late winter, the eventual acceptance of a rival of the opposite sex is a prelude to mating.

It is understandable that superstitions have arisen with regard to so well known a bird and associate of man as the robin. Indeed, it was said in Wales that a witch or the devil would possess a person who stole a robin's eggs; furthermore, the killing of a robin could, apparently, induce a swelling on the hand, bad handwriting or shaking of the hands in an adult. Again, epilepsy has been attributed to the destruction of a robin's nest by the patient and cows' milk is said to have become bloody because the farmer killed a robin (Armstrong, 1958a). Hence fits, ganglions, chorea or Parkinsonism in man, as well as mastitis in cows, have all been ascribed to killing a robin or taking its eggs.

Many territorial birds, especially the males, will fly at their reflection if they see it on a window-pane. At times, a robin will do this repeatedly, clearly assuming that a rival keeps on appearing. Whilst in country practice, an elderly woman told me that a robin had been tapping on her window, so she had not been surprised when her invalid husband died. Somehow, as a portent of death, a robin with its red breast has greater significance than if the tapping had been carried out by a pied wagtail *Motacilla alba* or a house sparrow *Passer domesticus*, neither of which birds have red in their plumage.

Like the robin, the swallow *Hirundo rustica* is a common bird and, moreover, it has a red throat. Swallows frequent farms where they breed in barns and, in the country, it has long been considered unlucky to interfere with one of their nests. The song of a swallow is a prolonged, musical twittering which is often hurried; for this reason, the bird has been associated with epilepsy as well as the robin (Armstrong, 1958b). In consequence, medicines made from bruised and macerated swallows were used as treatment for epilepsy, even as late as the 17th century.

As swallows arrive in Britain in springtime, so does the cuckoo *Cuculus canorus*. Announcing its own presence, at least the male, it is generally recognised as a bird with unusual abilities and habits. Yet, not only is 'cuckoo' a term for a silly person but psychotic and mentally ill patients are, or have been, dubbed 'cuckoo' as well, at least in general conversation. Furthermore, an old rural name for the cuckoo, particularly in Scotland, is 'gowk' and the same term is also used to indicate a fool. Then another dialect name for the cuckoo was 'gawk' which is used as well for an ungainly person and, again from the country, the word 'geck' signifies a cuckoo, a dupe or an object of scorn.

As is now well recognised, it needed the observations of Edward Jenner (1788), of vaccination fame, to show that it is the cuckoo which carries out the duping; the cuckoo can hardly be a geck when other bird species are tricked into slaving to rear its own offspring. The cuckoo, in its movements, flight and by its behaviour, is far from being awkward and is certainly neither a fool nor a dupe. Gawk, gowk and geck seem rather inappropriate country names for so specialised and successful a bird. Jenner described how a newly-hatched cuckoo ejected other eggs or nestlings from a dunnock's *Prunella modularis* nest: the young cuckoo tries to push anything which touches its lower back over the edge of the nest.

So the young cuckoo becomes the sole nest occupant and devours all food items brought by its foster parents. Before Jenner's day there was much uncertainty about the cuckoo's life history and it was thought that it was the adult cuckoo itself which would come and remove the remaining eggs or young from the nest. Some of this doubt may have arisen because the hen cuckoo, after she has laid her egg in the fosterer's nest, normally flies off with one egg of the clutch, which is then eaten. To illustrate the confusion at Jenner's time further, it appears that even the surgeon John Hunter asked Jenner (his pupil) to find him a cuckoo's nest (Fisk, D., 1959). In the past, the doctor lived closer to nature than is the case today, with the increasing size and spread of large towns. It is of interest that the coccyx was so named because of its resemblance to the form of a cuckoo's beak. Cuckoos are mobbed repeatedly by small birds in the breeding season and it is usually assumed that this is because of their resemblance to a bird-of-prey; of course, a cuckoo often flies like a raptor and has barred underparts like a sparrowhawk *Accipiter nisus*. However, to add to the difficulties, cuckoos are often mobbed by small birds of species which are not selected as fosterers and the cuckoo does not have a hooked beak. The cuckoo's beak is a decurved one like the coccyx, and adapted to seizing hairy caterpillars which are the bird's main, specialised item of food. The cuckoo problem is far from a final solution.

In rural lore, it used to be maintained that the cuckoo ate dirt, suffering, it would seem, from pica. Now the scientific name for the magpie is *Pica pica* but I hardly think that even this crafty bird readily eats dirt or earth. Naturally, like other crows, for instance the carrion crow *Corvus corone,* magpies readily seek carrion as well as searching for birds' eggs, fledglings, earthworms or other animal food. Amongst humans, pica is a curious habit, usually occurring under the age of 5 years; coal, paper, earth or sand may be craved for and ingested. In those cases I have come across, the disorder has disappeared without any special treatment; punishment by the parents only seems to make the condition worse.

All doctors receive biological training; some acquire more than a superficial interest in ornithology and this happens in all branches of medicine. Medical practice is based on the application of biological sciences, together with exact observation. Doctors and patients in the past have looked at birds, including their behaviour and a realisation of their beauty, in relation to the seasons. Now that medicine has become increasingly specialised and new scientific techniques are being introduced so frequently, it might be expected that doctors would seek relaxation in the watching and attempted understanding of the wildlife which remains around them. Those who live close to nature, both patients and doctors, are necessarily reducing in numbers because of urbanisation but surely it is preferable that the relationship between medical practice and birds, even if only very indirect at times, should continue. As the world population grows, so overall medical problems multiply and wildlife habitats shrink; for mental satisfaction and happiness, some balance must be achieved. The days of folklore remedies for disease have largely gone in Europe but human instincts and psychological requirements are still fundamentally the same. Some of these requirements need satisfaction through contact with the soil and life which is other than human. For some individuals, pot plants or gardens and perhaps keeping a caged bird or other less restrained pet, will suffice; others need a wilder environment and the sight and sounds of mammals and birds in their natural freedom.

### REFERENCES

ARMSTRONG, E. A. (1958a) The Folklore of Birds. London, Collins, pp. 167–168.

ARMSTRONG, E. A. (1958b) The Folklore of Birds. London, Collins, pp. 184–185.

JENNER, E. (1788) Observations on the natural history of the cuckoo. *Phil.Trans.Roy.Soc.London* **78**, 219–235.

FISK, D. (1959) Dr. Jenner of Berkeley. London.

